# Detailed genetic and functional analysis of the hDMDdel52/*mdx* mouse model

**DOI:** 10.1371/journal.pone.0244215

**Published:** 2020-12-23

**Authors:** Alper Yavas, Rudie Weij, Maaike van Putten, Eleni Kourkouta, Chantal Beekman, Jukka Puoliväli, Timo Bragge, Toni Ahtoniemi, Jeroen Knijnenburg, Marlies Elisabeth Hoogenboom, Yavuz Ariyurek, Annemieke Aartsma-Rus, Judith van Deutekom, Nicole Datson

**Affiliations:** 1 Department of Human Genetics, Leiden University Medical Center, Leiden, The Netherlands; 2 BioMarin Nederland BV, Leiden, The Netherlands; 3 Charles River Discovery Services, Kuopio, Finland; 4 Department of Clinical Genetics, Leiden University Medical Center, Leiden, The Netherlands; University of Minnesota Medical School, UNITED STATES

## Abstract

Duchenne muscular dystrophy (DMD) is a severe, progressive neuromuscular disorder caused by reading frame disrupting mutations in the *DMD* gene leading to absence of functional dystrophin. Antisense oligonucleotide (AON)-mediated exon skipping is a therapeutic approach aimed at restoring the reading frame at the pre-mRNA level, allowing the production of internally truncated partly functional dystrophin proteins. AONs work in a sequence specific manner, which warrants generating humanized mouse models for preclinical tests. To address this, we previously generated the hDMDdel52/*mdx* mouse model using transcription activator like effector nuclease (TALEN) technology. This model contains mutated murine and human *DMD* genes, and therefore lacks mouse and human dystrophin resulting in a dystrophic phenotype. It allows preclinical evaluation of AONs inducing the skipping of human *DMD* exons 51 and 53 and resulting in restoration of dystrophin synthesis. Here, we have further characterized this model genetically and functionally. We discovered that the *hDMD* and *hDMDdel52* transgene is present twice per locus, in a tail-to-tail-orientation. Long-read sequencing revealed a partial deletion of exon 52 (first 25 bp), and a 2.3 kb inversion in intron 51 in both copies. These new findings on the genomic make-up of the *hDMD* and *hDMDdel52* transgene do not affect exon 51 and/or 53 skipping, but do underline the need for extensive genetic analysis of mice generated with genome editing techniques to elucidate additional genetic changes that might have occurred. The hDMDdel52/*mdx* mice were also evaluated functionally using kinematic gait analysis. This revealed a clear and highly significant difference in overall gait between hDMDdel52/*mdx* mice and C57BL6/J controls. The motor deficit detected in the model confirms its suitability for preclinical testing of exon skipping AONs for human *DMD* at both the functional and molecular level.

## Introduction

Duchenne muscular dystrophy (DMD) is an X-linked, severe neuromuscular disorder with an incidence of 1:5000 newborn males due to genetic defects in the *DMD* gene, resulting in lack of functional dystrophin protein [1]. In skeletal muscle, dystrophin acts as a shock-absorber by connecting the actin cytoskeleton with the extracellular matrix. In addition to the structural role, as part of dystrophin-associated glycoprotein complex, it is thought to mediate cellular signaling between various proteins [2]. Its absence in DMD patients makes muscle fibers prone to contraction induced damage [3]. While no clinical signs are present at birth, the first disease symptoms typically begin at 2–3 years with proximal muscle weakness with diagnosis on average at 4 years of age [4]. Progressive loss of muscle tissue leads to wheelchair dependency around the age of 12, need for assisted ventilation around the age of 20, and most patients succumb to severe cardiomyopathy in the third-fourth decade of life [5].

The *DMD* gene is the largest known human gene. It contains 79 exons dispersed over 2.2 Mb genomic DNA, and forms an mRNA transcript of approximately 14 kb [6]. Various full-length and shorter isoforms exist that are expressed mostly in the nervous system, while the Dp427m isoform is expressed exclusively in skeletal, smooth and cardiac muscle [7]. Lack of Dp427m causes the most prominent clinical symptoms of DMD. However, lack of brain isoforms probably underlies cognitive deficits and behavioral issues observed in some patients [8]. More than 7000 mutations have been described for DMD patients [9,10]. These mutations consist of intragenic deletions of one or more exons (65%), duplications of one or more exons (6–10%), small mutations (most commonly point mutations) (25–30%) or other less frequent rearrangements (<1%).

One of the therapeutic approaches that is being explored for DMD is antisense oligonucleotide (AON)-mediated exon skipping [11]. AONs are chemically modified short synthetic RNA-like molecules that have the ability to interfere with the splicing process of pre-mRNAs in a highly sequence-specific manner. By inducing the skipping of an exon adjacent to a deletion of one or more exons, the disrupted reading frame can be restored [12]. This allows production of truncated but partly functional dystrophin proteins such as produced in Becker muscular dystrophy (BMD), a milder form of dystrophinopathy [13,14]. Although exon skipping is a mutation-specific approach, the majority of exon deletion breakpoints are clustered in a few specific introns, and the skipping of an adjacent exon applies to relatively large groups of patients, with exon 51 skipping applying to the largest group (13–14%) [15,16]. Two DMD exon skipping drugs (eteplirsen and golodirsen, for exon 51 and exon 53 skipping, respectively) have conditionally been approved by the US Food and Drug Administration (FDA) [17,18] based on slight increases in dystrophin levels in treated patients, while the company (Sarepta) has to show functional benefits of treatment by 2021 and 2024 respectively [19,20]. The European Medicines Agency (EMA) did not approve eteplirsen when reviewing the same results [21]. A third drug (viltolarsen for exon 53 skipping, developed by Nippon Shinyaku) has recently been approved by the Japanese Ministry of Health, Labour and Welfare and FDA [22,23].

Animal models are essential for preclinical drug development. For DMD, a number of animal models are available to evaluate the pathology of dystrophin deficiency and to test drug candidates [24]. The most widely used animal model is the *mdx* mouse having a nonsense point mutation in exon 23, which disrupts the production of full-length dystrophin [25]. While the *mdx* mouse has been useful by providing proof-of-concept for AON-induced exon 23 skipping *in vivo*, it has not allowed the testing of human-specific AONs because of species-specific sequence differences. Therefore, the hDMD transgenic mouse model, which contains the full-length human *DMD* gene integrated in a mouse autosome, was generated [26]. After crossing hDMD with *mdx* mice, it was observed that the human dystrophin expression compensated for the dystrophic phenotype caused by lack of mouse dystrophin [26]. As such, the hDMD/*mdx* mouse is not a disease model and can only be used to study whether human specific AONs can induce exon skipping at the mRNA level, and not to assess restoration of dystrophin expression, muscle quality or function. To address this, the hDMDdel52/*mdx* mouse, which carries an exon 52 deletion in the human *DMD* gene, was developed. Preliminary studies confirmed the resulting dystrophic phenotype of this mouse, with dystrophin restoration following treatment with human specific AONs inducing the skipping of *DMD* exon 51 or exon 53 [27]. Here, we follow up with an in-depth analysis of both the genotype and phenotype of this model. Notably, we observed additional, unexpected genetic rearrangements in the hDMD/*mdx* and hDMDdel52/*mdx* mouse models, with a tail-to-tail duplication of the *hDMD* gene, a small residue of exon 52, and an inversion of intron 51 in both copies. At a motor-functional level however, the dystrophin-deficiency of the hDMDdel52/*mdx* mice was confirmed by extensive MotoRater analysis revealing a clear kinematic gait deficit.

## Material and methods

### Mice and housing conditions

Different mouse strains were used to obtain the results described in this paper. The Leiden University Medical Center (LUMC) provided the B6.DBA2.129-hDMD^tg/tg^/LUMC*B10-Dmd^*mdx*^/J (hDMD/*mdx*) [26], hDMDdel52/*mdx* [27] and *mdx*/BL6 [28] mice. Here, mice were housed in individually ventilated cages (IVC) at 20.5°C with 12 hour dark-light cycles and fed regular RM3 chow (SDS, Essex, UK) *ad libitum*. All animal handling and experimentation performed at the LUMC was approved by the animal ethical committee under license AVD1060020171407.

hDMDdel52/*mdx* and *mdx*/BL6 breeding pairs were also shipped from the LUMC for breeding and experimental use at Charles River Laboratories (CR) (breeding: CR UK; experiments: CR Finland). Using these mice, CR generated their own colony of hDMDdel52/*mdx*, in addition to C57BL6/J controls. At CR Finland, mice were housed in IVCs at a density of four-five mice per cage in a temperature- and humidity-controlled environment (22 ± 1°C, humidity levels 30–70%) with a normal light-dark cycle (7:00–20:00). Food (Purina Lab Diet 5001) and water was available *ad libitum* to the mice in their home cages. Experiments were executed as specified in the license authorized by the national Animal Experiment Board of Finland and according to the National Institutes of Health (Bethesda, MD, USA) guidelines for the care and use of laboratory animals Experiments performed at the LUMC were approved by The Animal Experiments Committee (DEC). Effort was put in minimizing the burden and distress caused to the animals as much as possible.

### MotoRater analysis

#### Fine motor and gait kinematic analysis

Fine motor and gait kinematic analysis experiments were carried out at CR (Finland). Fine motor skills and gait properties were assessed at the age of 6, 14 and 20 weeks in 10 hDMDdel52/*mdx* mice and 10 C57BL6/J controls (mixed genders) using the walking mode of a high precision kinematic analysis method (MotoRater, TSE Systems, Homburg, Germany). Before the test sessions, mice were marked at appropriate points of the body to ease the data analysis process. The movement data were captured using a high-speed camera (300 frames/second) from three different dimensions (below and both sides). The captured videos of each mouse were first converted to SimiMotion software to track the marked body points *i*.*e*. to have raw data (coordinates) about the movement of the different body points in relation to the ground. Each of the three dimensions was correlated. Different gait patterns and movements were analyzed using a custom-made automated analysis system. The analyzed parameters included *e*.*g*.: 1) general gait pattern parameters (stride time and speed, step width, stance and swing time during a stride, interlimb coordination), 2) body posture and balance (toe clearance, iliac crest and hip height, hind limb protraction and retraction, tail position and movement), and 3) fine motor skills (swing speed during a stride, jerk metric during swing phase, angle ranges and deviations of different joints, vertical and horizontal head movement). The analysis provided altogether 95 different parameters related to fine motor capabilities and gait. Data were analyzed for distinctive parameters, as well as using principal component (PC) analysis for the acquired parameters.

### Tissue collection

One week after the MotoRater analysis at the age of 21 weeks the mice were euthanized by deep anesthetization with sodium pentobarbital (60 mg/kg Mebunat, Orion Pharma, Finland) and were subsequently subjected to cardiac puncture. Then, the mice were transcardially perfused with PBS in order to remove blood from the tissues and a variety of different tissues were collected including skeletal muscle (gastrocnemius, tibialis anterior, quadriceps), heart, diaphragm, aorta, liver, kidney and lung. Tissues were immediately snap frozen by immersing in isopentane au bain marie in liquid nitrogen, placed in cryovials prechilled on dry ice and stored at -80°C until further use.

### Dystrophin analysis

#### Protein lysate preparation from hDMDdel52/*mdx* heart

Protein lysates were prepared from snap frozen hDMDdel52/*mdx* mouse hearts by sectioning or cutting off small pieces of in total ~ 5–10 μg, placing them in a MagNA Lyser vial (without beads) and adding 200 μl protein lysis buffer (15% SDS, 75 mM Tris-HCl pH 6.8, one protease inhibitor cocktail tablet (Roche/Sigma 04693159001)/8 ml; 5% β-mercaptoethanol). After briefly spinning down, approximately 25 ceramic beads (MagNA Lyser Green Beads; Roche 03358941001) were added to each tube. The samples were then homogenized by 2–4 cycles (20 seconds; 7000 rpm) in the MagNA Lyser Instrument (Roche 03358976001) and spun down for 5 min at 13,000 rpm. The supernatant was supplemented with glycerol (final concentration 20% v/v) and then samples were stored at -80°C until further use.

To measure total protein concentration, 20x dilutions of the lysates were measured using the Pierce 660 nm protein assay (#226607, Thermo Scientific) with added ionic detergent compatible reagent (#22663, Thermo Scientific), according to the manufacturer’s instructions.

#### Dystrophin analysis in heart using capillary Wes immunoassay

Wes analysis was performed as previously described [29] on a Wes system (#004–600, ProteinSimple) according to the manufacturer’s instructions using a 66–440 kDa separation module (#SM-W008, ProteinSimple) combined with a no secondary detection module (#DM-003).

For dystrophin detection in tissues, a 1/50 dilution of mouse monoclonal Mandys106 (kindly provided by Dr. Glenn Morris) was used, which recognizes an epitope encoded by exon 43 of human dystrophin. To detect Mandys106, HRP-labeled antibody from ProteinSimple (#042–205, anti-mouse secondary antibody) was used. Mouse heart samples were diluted to an appropriate concentration (250 μg/ml, which resulted in a loaded amount of 1.25 μg per well/capillary) in sample buffer (100x diluted ‘10x Sample Buffer 2’ from the separation module), then mixed with fluorescent master mix and heated at 95°C for 5 min. The samples, blocking reagent (antibody diluent), primary antibodies (in antibody diluent), HRP-conjugated secondary antibodies and chemiluminescent substrate were pipetted into the plate (part of separation module) and a Wes run was performed using instrument default settings.

To control for differences in signals between experiments, a 6-point calibration curve of a hDMD/*mdx* mouse muscle sample (obtained from the LUMC), ranging from 0.004–1.0 μg, was routinely included for dystrophin detection. This reference sample was selected from a panel of hDMD/*mdx* mice based on it displaying average dystrophin levels. Wes runs were considered valid if the linearity of the calibration curve displayed an R^2^ > 0.99, based on 4–6 points in a relevant concentration range.

In addition, to correct for loading differences, vinculin levels were also determined by Wes in the diluted samples. Vinculin is commonly used for normalization and expressed in most tissues, with high levels expressed in muscle tissue. A 1/100 dilution of anti-vinculin antibody (E1E9V rabbit monoclonal; #13901S Cell Signaling) followed by ProteinSimple anti-rabbit secondary antibody (#042–206) produces very strong ~125 kDa peaks in Wes. Since vinculin abundance was too high to get reliable chemiluminescence values (substrate depletion), we spiked/diluted the standard ProteinSimple secondary anti-rabbit-HRP antibody with 1/1000 unconjugated anti-rabbit antibody (ab6702, Abcam) to reduce HRP activity. The vinculin calibration curve of the same hDMD/*mdx* mouse used for dystrophin analysis ranged from 0.125–4.0 μg. The final vinculin-corrected dystrophin values were generated by first expressing both the dystrophin and vinculin signals as percentage of the hDMD/*mdx* control (% hDMD; using a calibration curve) using the formula below and then dividing the dystrophin % hDMD value by the vinculin % hDMD value:

$$\%\;hDMD=\frac{sample\;protein\;equal\;to\;x\;\mu g/ml\;hDMD\;(from\;cal.curve)x\;\mu g/ml\;sample\;loaded}{x\mu g/ml\;sample\;loaded}.$$

### Genomic DNA isolation and genotyping of deletion 52 region by TaqMan analysis

Genomic DNA (gDNA) was isolated from 10–20 mg kidney, liver or quadriceps with DNAeasy blood and tissue kit (#69506, Qiagen) according to the manufacturer’s protocol. In short; samples were lysed overnight in 56°C incubator with 180 μl buffer ATL + 20 μl proteinase K. When lysis was complete 200 μl AL buffer was added, vortexed and 200 μl ethanol (96–100%) was added. Samples were loaded on the columns, centrifuged and washed twice. gDNA was eluted in 150 μl elution buffer and measured by Nanodrop One (ThermoFisher), followed by dilution to 10 ng/μl for qPCR and to 25 ng/μl for droplet digital PCR (ddPCR) analysis.

TaqMan assays were designed to detect human specific *DMD* sequences for intron-exon or exon-intron junctions from exon 51–53 (For details see S1 Table). For assay A, B and D, a separate master mix was prepared with 15% excess in quadruple per sample with 5 μl gene expression master mix (#4369510, ThermoFisher), 0.5 μl FAM labeled hDMD TaqMan assay, 0.5 μl VIC labeled TaqMan copy number reference assay against mouse transferrin receptor gene (*Tfrc*) (#4458366, ThermoFisher) and 2 μl H_2_O. Eight microliter master mix was aliquoted per well of a 384 well plate, 4 wells per sample for each assay. Two microliter gDNA (10 ng/μl) was added to each well containing a master mix. The plate was centrifuged and ran in a ViiA7 thermal qPCR cycler (ThermoFisher) using the following program: 2 min at 50°C, 10 min at 95°C and followed by 40 cycles of 15 s at 95°C, 1 min at 60°C. On each plate one *mdx*/BL6 (from CR bred colony) and at least one heterozygous hDMD/*mdx* (from LUMC) reference gDNA sample were taken along for negative controls and references respectively.

The qPCR threshold for Cq determination was set in the exponential phase equally for all assays. Data was exported as a.txt file and imported in the CopyCaller software v2.1 (freeware from ThermoFisher). In the analysis settings, one heterozygous hDMD/*mdx* sample was selected as a reference and the reference copy number was set to 1 copy. Assays were selected in the assay selection window and data was exported. The exported file showed the calculated and predicted copy number per assay per sample and its z-score. Calculation was based on the 2^-ΔΔCq^ [30] method normalized by the reference target *Tfrc*.

### Droplet digital PCR

ddPCR for quantification of *hDMD* sequence targets was performed, as previously described [31], in duplicate per sample using the same TaqMan assays as used in qPCR described above with addition of assay C (S1 Table) and assay E and F (S2 Table). In addition, TaqMan assays were also designed to detect human specific *DMD* sequences for intron-exon or exon-intron junctions from exon 73–79 3’UTR (S1 Table). Each assay was multiplexed with VIC labeled TaqMan copy number reference assay against mouse *Tfrc* as in the qPCR described above. Two microliter gDNA (25 ng/μl) was used as input per sample duplicate. Data was analyzed with QuantSoft analysis pro (Bio-Rad) and the threshold was set between negative and positive droplets for each dye-channel separately. Measured concentrations of *Tfrc* represents two copies of this target (located on chromosome 16) and was used to normalize *hDMD* target copy numbers. Due to the absolute quantification of ddPCR, reference samples for normalization were not needed. For the quantification of intron 51 inversion event among *hDMD* constructs, EvaGreen-based ddPCR was performed using inversion-specific primers (S1 Table). With their known copy numbers, *hDMD* exon 1 and Myostatin (*Mstn)* specific primers were used as internal and external controls respectively. ddPCR was carried out in 20μl reaction mix including 10μl 2x ddPCR EvaGreen supermix, 0.2μl forward and reverse primer (10μM each) and 2 μl genomic DNA (25ng/μl) isolated from hDMDdel52/*mdx* (2 copies), hDMDdel52/*mdx* (4 copies) and hDMD/*mdx* (all obtained from the LUMC). The plate was sealed and the following PCR program was run: 5 min initial denaturation, 40 cycles of 95°C (30s), 60°C (60s), followed by signal stabilization at 4°C (5 min) and 95°C (5 min). After thermal cycling, the plate placed in the QX200 Droplet Reader to read the droplets. After completion of the run, the data was analyzed in QuantaSoft Software (Bio-Rad). Normalization was done relative to *hDMD* exon 1 copy number.

### Sequence analysis of hDMDdel52-53 mice

A 666 bp PCR fragment over the breakpoint was generated with primer pair i51F8.1: 5’-CAGTGTGCGGTCTAGTGGAA-3’ and i53R4.2: 5’-CAGTTGACCATAAATGCAAAGG-3’. This fragment was extracted from gel with QIAquick Gel Extraction Kit (Qiagen #28115) according to manufacturer’s protocol. For sequence analysis, 25 μM primer with 100ng PCR product was supplemented with ddH2O till 20 μl. Sequence analysis was done by BaseClear B.V. (Leiden, The Netherlands) and results were analyzed in SnapGene. hDMDdel52-53 samples were obtained from the CR colony.

### Sequence analysis of exon 52 with flanking regions

The gDNA, obtained from LUMC bred hDMDdel52/*mdx* mice, was prepared for long fragment capture following the SureSelect^XT^ (v3, Agilent Technologies) target enrichment manual with some modifications. The gDNA was sheared using Covaris g-TUBES (#520079, Covaris) to 8 kb according to the manufacturer’s guidelines. To remove fragments smaller than 4 kb a size selection was performed using the BluePippin (Sage Science) following the manufacturer’s guidelines. The DNA was then subjected to an end repair and TruSeq^™^ (v2, Illumina) adapter ligation using Kapa Hyper prep library kit following the manufacturer’s instructions. The pre capture PCR was performed using the universal PCR primers for Illumina with Takara’s LA PCR kit (v2.1). Concentration and fragment sizes were determined with a Qubit fluorometer and Agilent’s Femto Pulse System according to the manufacturer’s instructions throughout the experiments. The sample was hybridized and captured according to the SureSelect^XT^ Target Enrichment for Illumina Paired-End Multiplexed Sequencing protocol (vB.1, 2014, 16 h hybridization) using *hDMD*-specific DNA probes (probe design number: 0259601) [32]. The target fragments were amplified in the post capture PCR using Takara’s LA PCR kit (v2.1). Subsequently the sample was prepared for sequencing using Oxford Nanopore library prep kit (LSK109). The library was then loaded on an Oxford Nanopore’s Minion chip (R9.4.1) following the instructions of the vendor and run for 48 h. After the run was completed, Guppy (v3.2.4) was used for accurate base calling using the default settings. After cleaning up the data for adapters using PoreChops (v0.2.4), the reads were mapped using Minimap2 (v0.2-r123) against a custom genome, namely the entire mouse reference genome (GRCm38/mm10) with additional chromosomal sequence for the entire human *DMD* region and the SV40 region. For detecting structural variance, Sniffles (v1.0.11) was used in the default settings. In addition to Oxford Nanopore long read massive parallel sequencing, to elucidate the exon 52-inversion and inversion-sv40 poly(A) junctions, specific fragments were generated by inversion specific primers (the same primers as in EvaGreen-based ddPCR). PCR products were purified prior to Sanger sequencing using AMPure XP purification kit (A63882 Beckman Coulter). After purification, concentration of PCR products was measured by Qubit 4 Fluorometer (ThermoFisher) and 20 ng/μl of each sample was sent to Sanger sequencing.

### Fluorescent *in situ* hybridization

For the FISH experiment, 200 μl blood was collected from the tail-vein of hDMD/*mdx*, hDMDdel52/*mdx* and *mdx*/BL6 males (LUMC bred colonies) using lithium-heparin coated tubes. Blood samples were cultured for 72 hours in PB-MAX Karyotyping Medium (ThermoFisher Scientific, Waltham, MA, USA) at 37°C and 5% CO_2_, to stimulate culture of T-lymphocytes from the whole blood. The hDMD/*mdx* and hDMDdel52/*mdx* mice were homozygous for the transgene. Cells were harvested and fixed in methanol/acetic acid (3:1) by gentle resuspension. The fixed samples were stored in -20°C until use. For each strain, a drop of sample was placed on a drop of 45% acetic acid and spread on clean microscope slides and air dried, washed three times for 10 min with phosphate buffer saline (PBS), then dried by baking at 65°C for 2 h followed by 2 h incubation in 2xSSC (2xSSC = 0.3 M NaCl, 0.03 M sodium-citrate, pH 7.0). Samples were dehydrated by adding 70, 90 and 96% ethanol series and air dried again. After dehydration, the slides were treated with RNase A for 1 h and followed by denaturation of the slides in 70% formamide and 2xSSC for 2 min at 74°C, then dehydrated in ethanol. In parallel, probes were denatured at 74°C for 5 min. Hybridizations were carried out overnight in a moist dark chamber at 37°C in 50% formamide, 2xSSC and 10% dextran sulphate using the following probes; 1 μl RP11-151J4 (locus Xp21.1, label FITC, targeting exon 2), 1 μl RP11-609C15 (locus Xp21.2, label TRITC, targeting intron 62–77) and 3 μl hybridization mix (50% formamide/2xSSC/10% dextran sulphate). The slides were washed two times for 5 min in 2x SSC/0.1% Tween-20 (pH 7.0) at 42°C, followed by two times washing for 3 min in 0.1xSSC at 60°C and dehydration. Slides were counterstained with 4′,6-diamidino-2-phenylindole (DAPI; 0.25 μg/ml) and propidium iodide (2.5 μg/ml) in Vectashield (Vector) and viewed under the fluorescent microscope (Zeiss Axio D2) and images were taken using image capture (CytoVision, Leica Biosystems).

## Results

### Unexpected dystrophin expression in hDMDdel52/*mdx* mice

**Fig 1:**
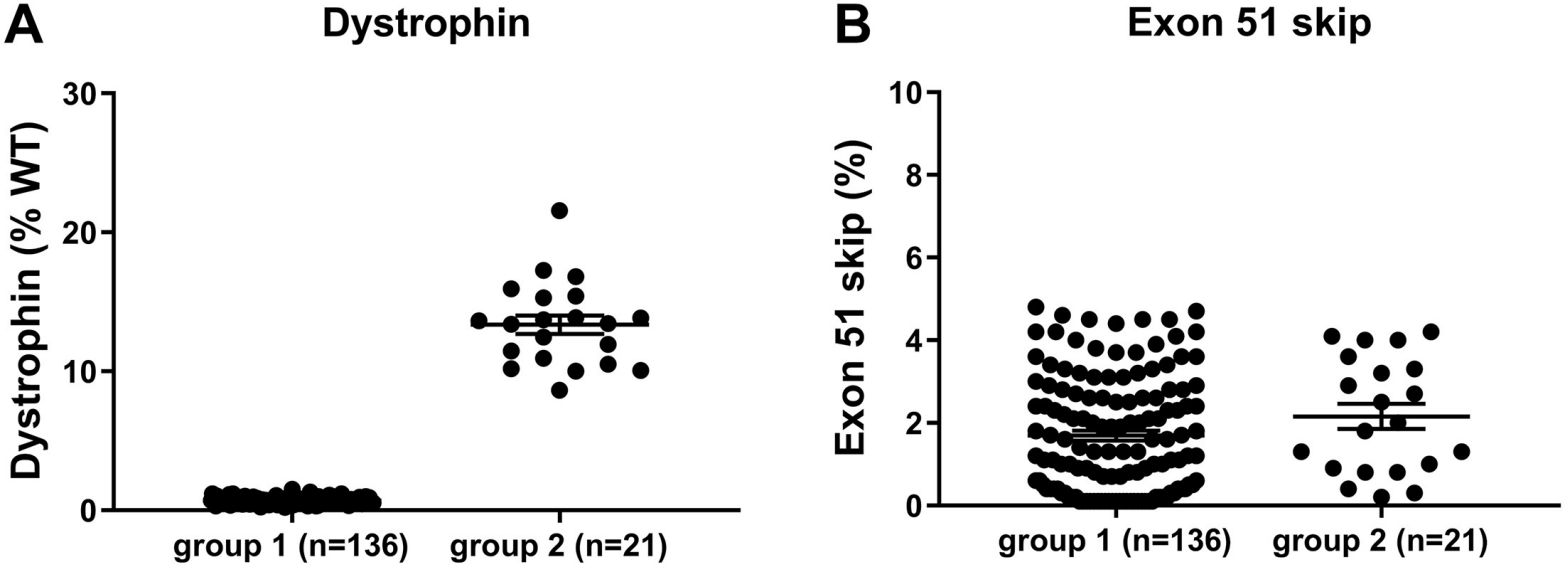
Unexpectedly high dystrophin levels detected in the heart of untreated and AON treated hDMDdel52/*mdx* mice. A. Wes capillary immunoassay analysis revealed two groups of mice. Most mice had very low dystrophin levels (0.2–1.5%, mean: 0.7, standard deviation (SD): 0.2, group 1), while some treated and untreated mice had dystrophin levels in the range of 8.6–21.6% (mean: 13.4, SD: 3.0, group 2). B. Droplet digital qPCR revealed that exon skipping levels were comparable between the groups and therefore could not account for the differences observed in dystrophin protein levels. (Group 1: 0.1–4.8%, mean: 1.7, SD: 1.4 group 2: 0.2–4.2%, mean: 2.2, SD: 1.4). WT; wild type.

Dystrophin quantification of skeletal muscle and heart lysates of hDMDdel52/*mdx* mice was performed using capillary Wes immune assay (Wes). This revealed that, in spite of the exon 52 deletion, some untreated mice were identified as dystrophin positive in the colony, with levels of >10% of normal. In addition to that, some mice treated with exon 51 skipping AONs had unexpectedly high dystrophin levels. The most striking difference was observed in the heart where high dystrophin levels did not correlate with exon skipping levels which eliminates confounding AON effects (Fig 1). Two groups of mice were formed: defined as group 1 (low dystrophin; 0.2–1.5%) and group 2 (high dystrophin; 8.6–21.6%), containing 136 and 21 mice respectively. Droplet digital qPCR revealed that exon skipping levels were comparable between the two groups and therefore could not account for the differences observed in dystrophin protein levels observed. In skeletal muscle, some of the mice in group 1 also showed relatively high dystrophin levels, but this was due to the treatment with an efficacious AON (the mean values for dystrophin in quadriceps were 4.2% for group 1 and 39.9% for group 2).

### Group 2 mice express dystrophin due to a secondary mutation in the *hDMD* gene

**Fig 2:**
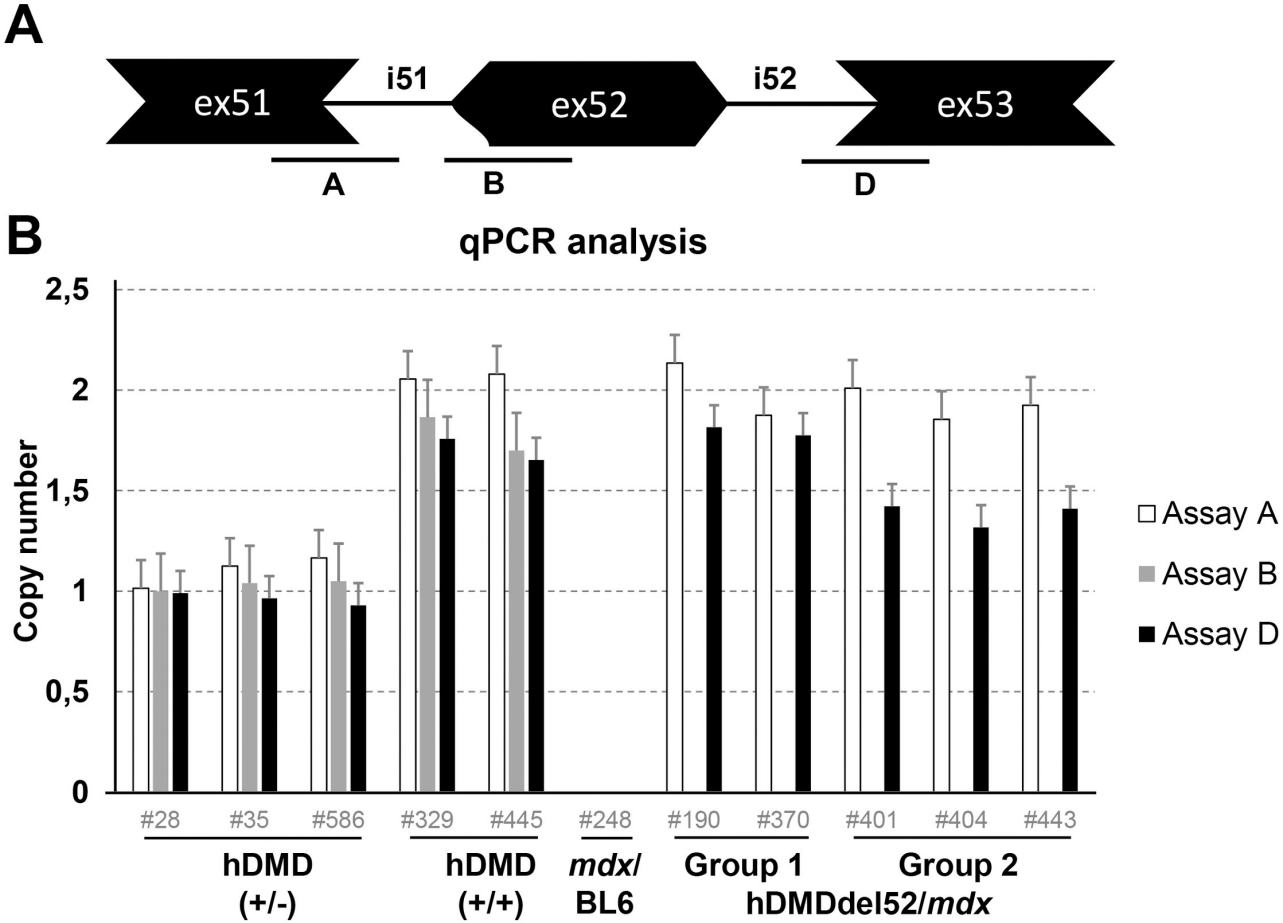
qPCR analysis of exon 51, 52 and 53 copy numbers. A. Schematic representation of the probe locations. B. qPCR analysis of hDMD/*mdx* (+/- and +/+), *mdx*/BL6 and hDMDdel52 (group 1 and group 2) mice. To detect whether the hDMDdel52/*mdx* group 2 mice had an additional deletion of exon 51 or 53, qPCR was performed with primers targeting exon 51, 52 and 53 exon-intron boundaries. In both homozygous and heterozygous hDMD/*mdx* mice all exons could be detected, as expected with a 2-fold difference in copy number. For group 1 mice, exon 51 and exon 53 were detected, while exon 52 was absent. For the group 2 mice however, a reduced signal for exon 53 was detected.

A possible explanation for the unexpectedly high dystrophin expression in group 2 mice, could be a secondary mutation that restored the reading frame. For a deletion of exon 52, the reading frame can be restored by an additional deletion of either exon 51 or 53. To explore this further, qPCR analysis was performed on genomic DNA of hDMDdel52/*mdx* group 1 and 2 mice and of hDMD/*mdx* and *mdx/*BL6 controls, using primer pairs targeting exon-intron boundaries for human exon 51 (assay A), exon 52 (assay B) and exon 53 (assay D) (Fig 2A). In the hDMD/*mdx* mice all measured exons were detected with twice the copy number in homozygous compared to heterozygous hDMD/*mdx* mice as expected. All hDMDdel52/*mdx* mice lacked the signal for the exon 52 assay (assay B), confirming deletion of this region. However, for the exon 53 assay (assay D) a lower signal was obtained for the exon 53 assay (assay D) in group 2 mice, suggestive of a deletion of exon 53. Strikingly, the copy numbers of assay D were not approximately 1, which would be expected if exon 53 was deleted on one of the alleles, but closer to 1.5 (Fig 2B).

**Fig 3:**
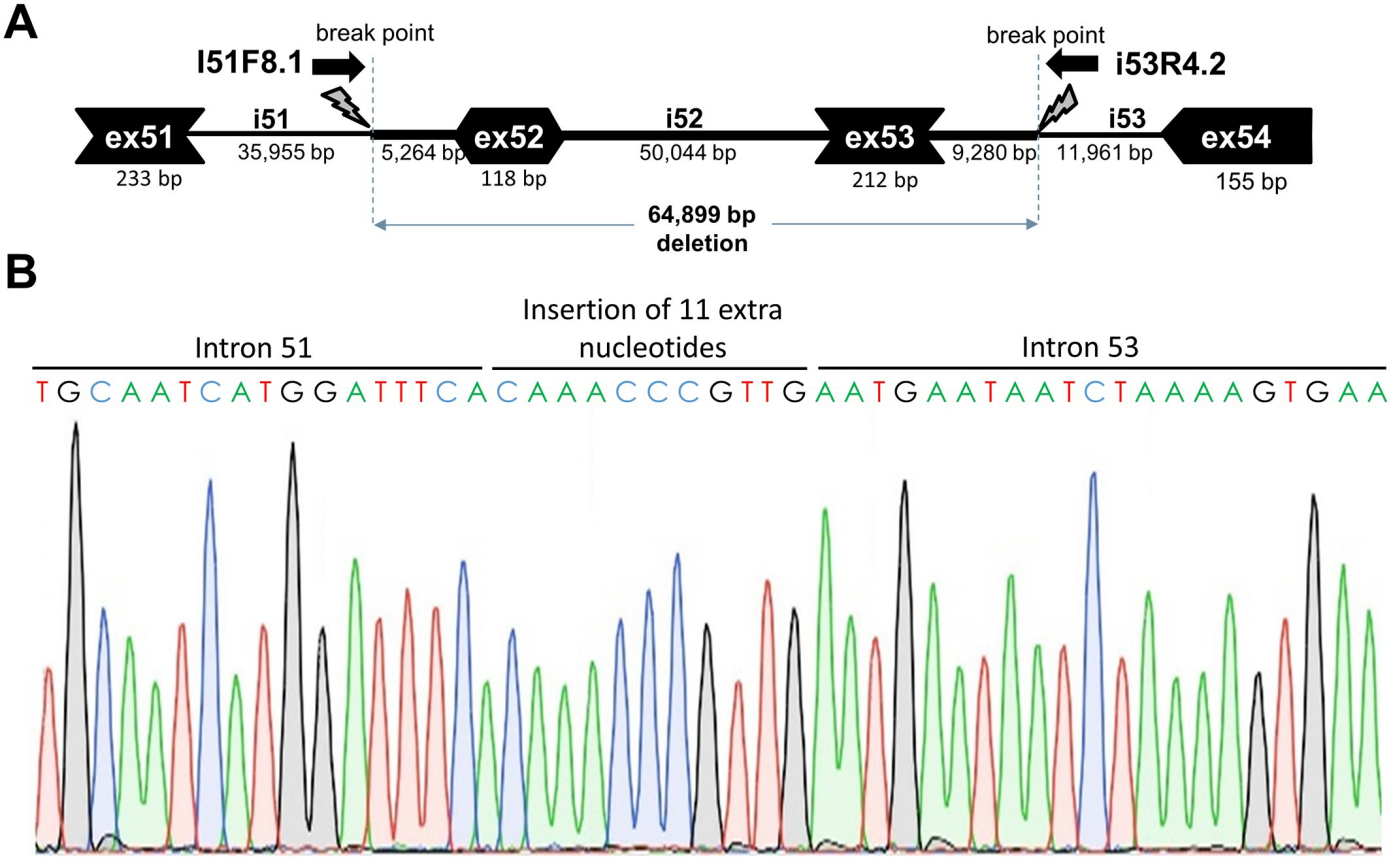
Deletion breakpoint and sequence analysis. A. Schematic depiction of the ~65 kb genomic deletion. B. Sanger sequence trace of the deletion breakpoint, including an insertion of 11 nucleotides at the break point.

To pinpoint the breakpoint resulting in the additional deletion of exon 53 in the group 2 mice, a ddPCR walk was done using several primer pairs located at intervals of approximately 5 kb throughout introns 51 and 53. The deletion breakpoint could thus be narrowed down based on assessment of copy numbers of the amplicons shifted from 2 to 1.5. Subsequently more primer pairs were developed at closer intervals, ultimately pinpointing the deletion breakpoint to an approximately 700 bp fragment generated with forward primer i51F8.1 located in intron 51 approximately 5 kb upstream of exon 52 and reverse primer i53R4.2 located in intron 53 approximately 9 kb downstream of exon 53 (Fig 3). Sequencing of this fragment elucidated the deletion breakpoint and indicated a ~65 kb genomic deletion and an insertion of 11 nts in these mice, which was not present in the low-dystrophin group 1 mice. This reading frame restoring deletion of exon 53 could be traced back to a single founder mouse.

### Copy number differences in the hDMD, hDMDdel52/*mdx*, and hDMDdel52-53/*mdx* mice

**Fig 4:**
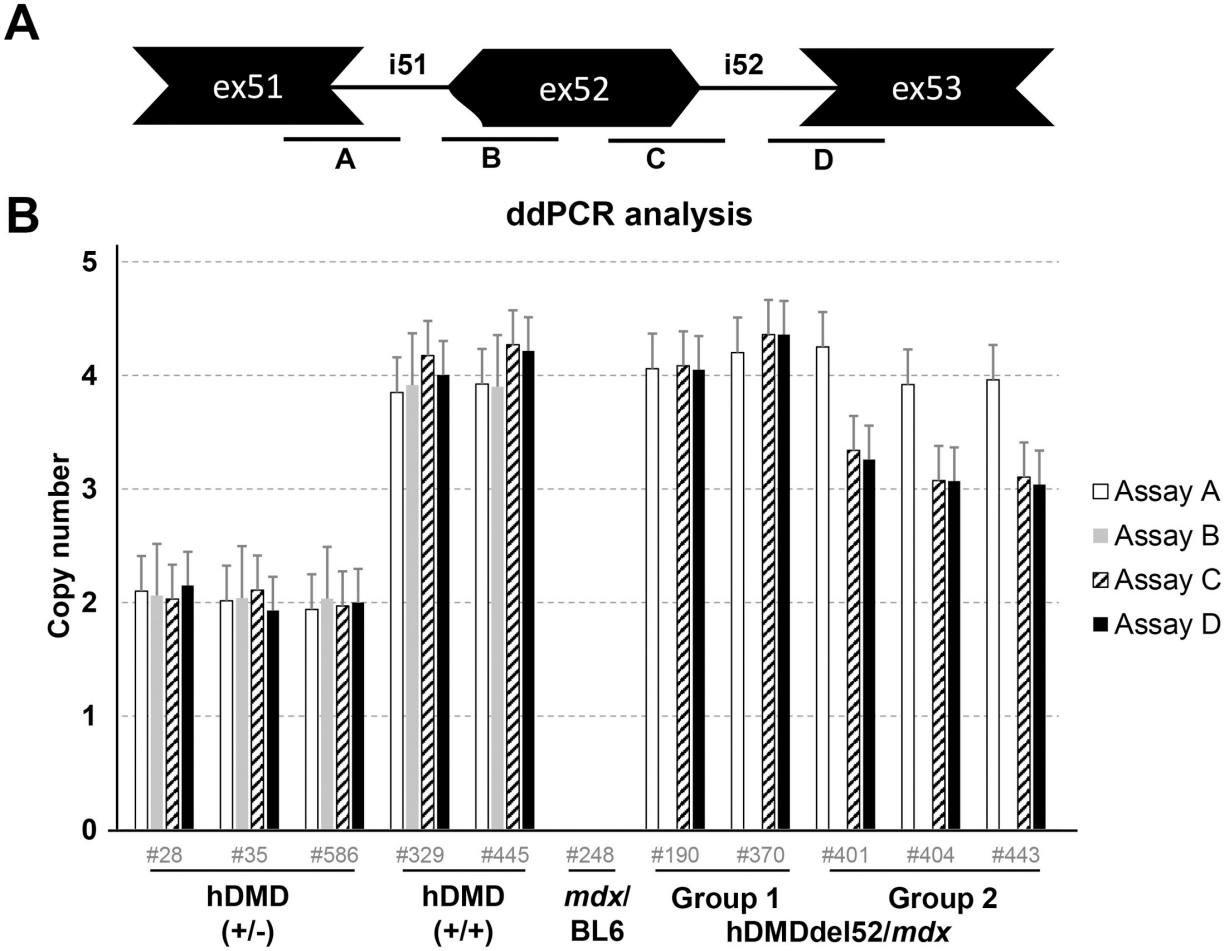
ddPCR copy number analysis. A. Schematic representation of the probe locations. B. ddPCR analysis of copy numbers of exons 51, 52 and 53 in hDMDdel52/*mdx* and hDMDdel52-53/*mdx* mice and controls, relative to *Tfrc* copy number. Most assays showed a double signal for both hDMD/*mdx* and hDMDdel52/*mdx* mice, while no signal was detected in *mdx*/BL6 mice. For the hDMDdel52/*mdx* and hDMDdel52-53/*mdx* mice, the probe on the intron 51- exon 52 boundary (assay B) did not give a signal. However, the probe on the exon 52 –intron 52 boundary did (assay C). As previously observed, assay C and assay D showed lower signals in hDMDdel52-53/*mdx* mice compared to hDMDdel52/*mdx*.

Having confirmed that there was indeed a genomic deletion of exon 53, the group 2 mice were henceforth referred to as hDMDdel52-53/*mdx*. We delved deeper into the aberrant copy numbers we had observed in these mice. For the absolute quantification, ddPCR analysis was performed by extending the previous qPCR analysis on genomic DNA with an additional assay at the 3’ end of exon 52 (Fig 4A, assay C). *Tfrc* copy number was used as a reference, assuming that this autosomal gene is present in the mouse genome in 2 copies. Interestingly, copy numbers of assays A, B, C and D were twice the expected number in homozygous and heterozygous hDMD mice when compared to the autosomal *Tfrc* reference gene (2 and 4, rather than the expected 1 and 2) (Fig 4B). The most likely explanation for these findings is that two copies of the *hDMD* construct are integrated per mouse chromosome 5. In the hDMDdel52/*mdx* mice assays A and D confirmed the two hDMD copies. However, given the anticipated deletion of exon 52 no signal was expected for both assays B and C. Instead assay C was positive and suggesting two copies as well. Presumably, the genomic exon 52 deletion was partial on both copies. In the hDMDdel52-53/*mdx* mice, assays C and D were unexpectedly positive given the assumed deletion of exons 52 and 53, but at three copy numbers. This would point to the genomic exon 52/ exon 53 deletion to be present in one copy only, leaving the other three copies intact.

### hDMD/*mdx* and hDMDdel52/*mdx* mice have the *hDMD* gene in a tail-to-tail duplication

**Fig 5:**
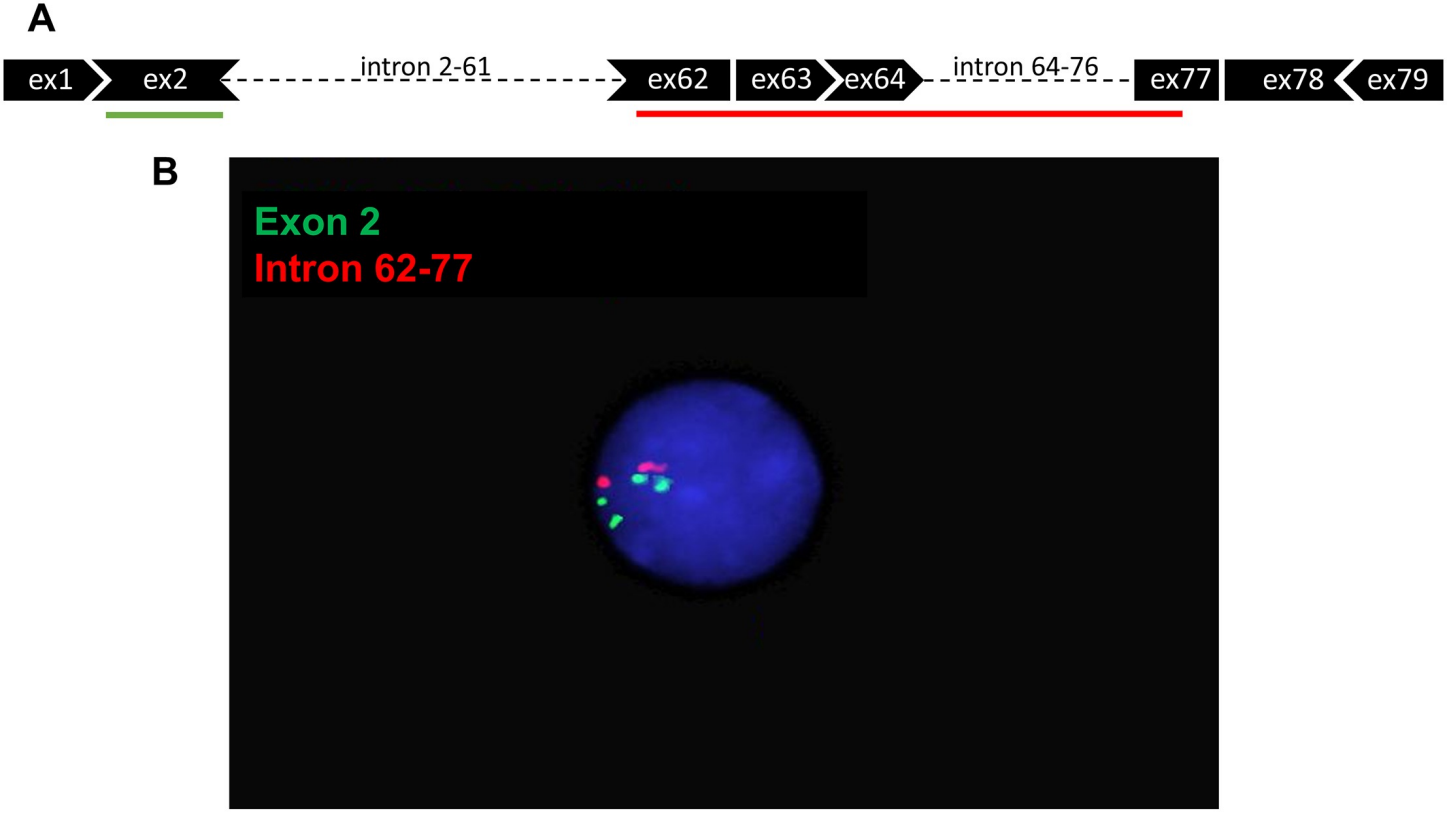
Detection of *hDMD* tandem duplication in hDMDdel52/*mdx* mouse by FISH analysis in interphase nuclei. A. Schematic representation of FISH probes. B. Hybridization results under fluorescent microscopy. Two signals can be seen for the red probe which targets intron 62–77, while four signals are detected for the green probe targeting exon 2. Because metaphase spreads could not be detected, interphase nuclei were used for the analysis. hDMDdel52-53/*mdx* mice were not included in this analysis.

**Fig 6:**
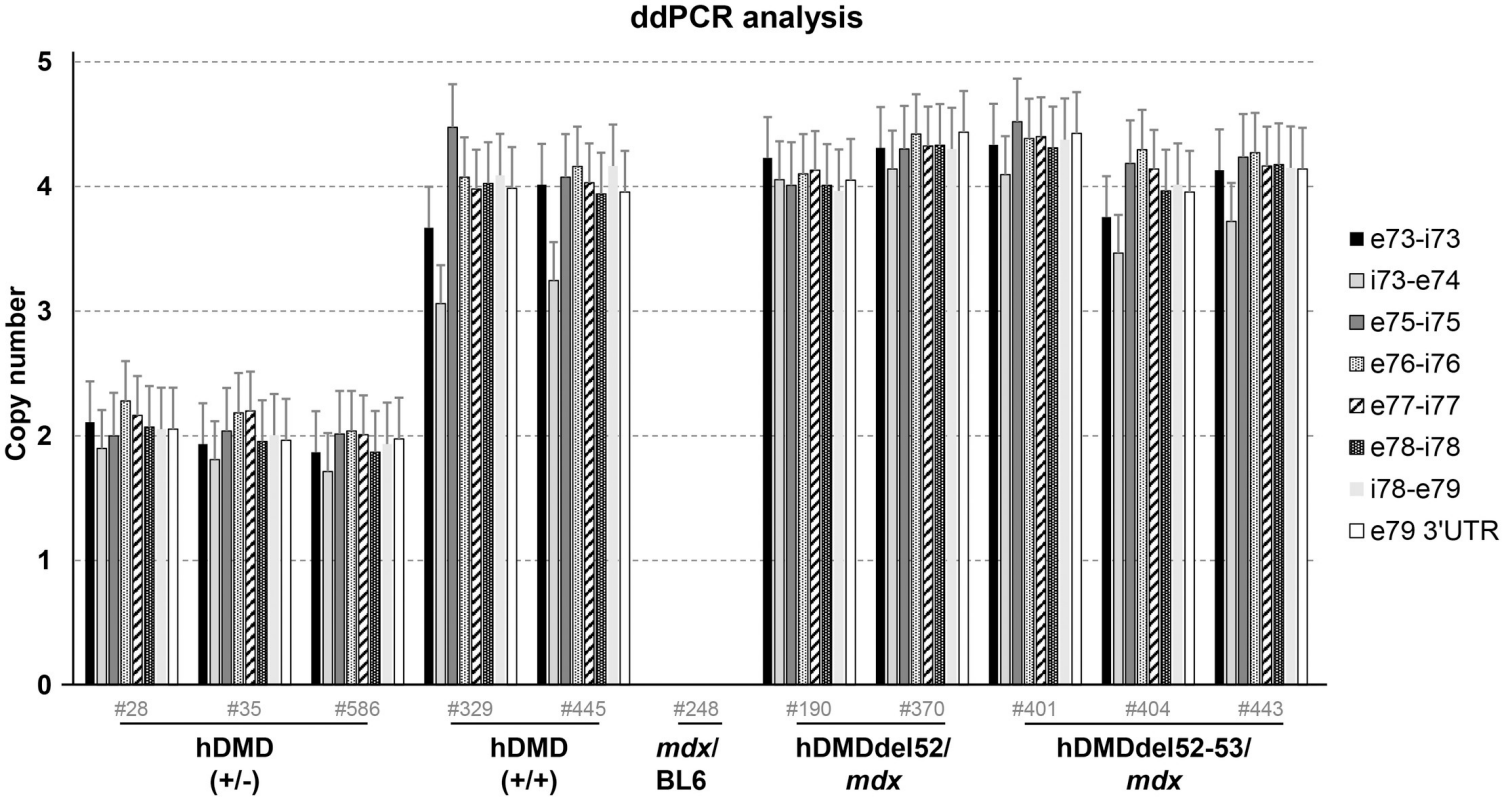
ddPCR analysis of copy numbers of exons 73 to 79 in hDMDdel52/*mdx* and hDMDdel52-53/*mdx* mice and controls, relative to *Tfrc* copy number. Probes gave a double signal for hDMD, hDMDdel52/*mdx* and hDMDdel52-53/*mdx* mice, while no signal was detected in *mdx*/BL6 mice.

To confirm a hDMD duplication in the hDMD/*mdx* and hDMDdel52/*mdx* mice, a FISH analysis was performed using human specific BAC probes targeting *DMD* exon 2 and its flanking introns (green) and one spanning *DMD* intron 62 to intron 77 (red) (Fig 5A). As metaphase spreads could not be detected, we were only able to analyze interphase nuclei (Fig 5B). Fifty nuclei could be analyzed for hDMD/*mdx*, hDMDdel52/*mdx* and *mdx*/BL6 mice. No signal was detected for *mdx*/BL6 mice, as expected because the probes were specific for human *DMD* sequences. The nuclei from hDMD/*mdx* and hDMDdel52/*mdx* mice contained four green and two red dots each: two signals per locus for the probe targeting exon 2, and one signal per locus for the probe targeting intron 62–77 (homozygous animals). The most likely explanations for this observation are: 1) a tail-to-tail duplication, which gives rise to merging of two red signals for each locus, or 2) a partial duplication of the *hDMD* construct, where only the 5’ part of the gene was duplicated. Based on our ddPCR analysis (Fig 2) we knew that at least the region up to exon 53 was duplicated. To address the possibility of a partial duplication we performed ddPCR quantification of exons 73–79, and did not find evidence for a deletion in this region (Fig 6). Therefore, the most likely explanation for our observations is a tail-to-tail duplication, which probably occurred during the generation of the hDMD/*mdx* mice.

### Genetic analysis of exon 52 and flanking introns

**Fig 7:**
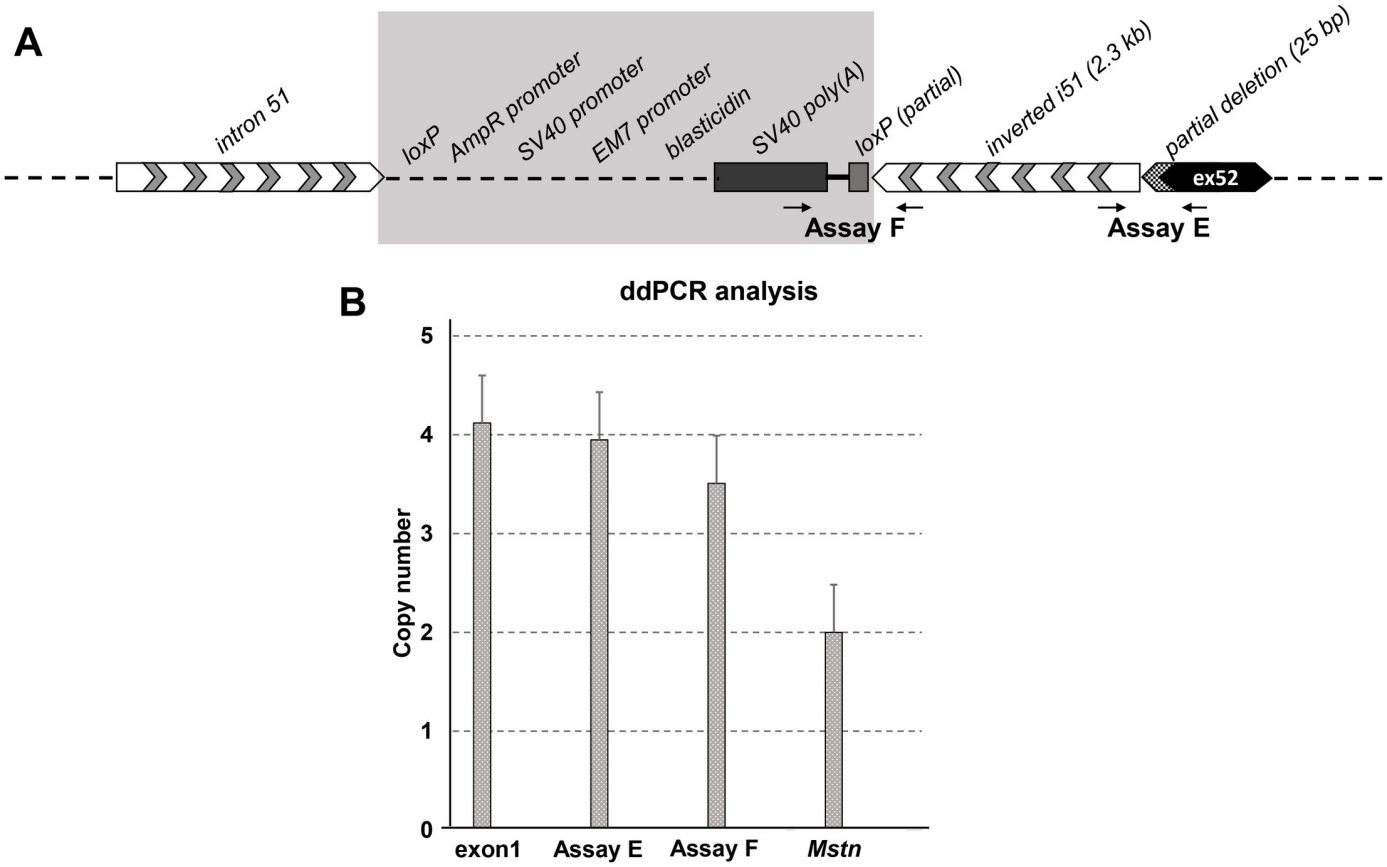
Genetic analysis of exon 52 deleted region. A. Schematic representation of the exon 52 deletion region with flanking sites; the 25 bp partial deletion of exon 52 and 2.3 kb inversion of intron 51 were confirmed by sequencing. The map of the shaded area could not be confirmed by sequencing. B. ddPCR analysis of intron 51 inversion events in *hDMD* constructs. hDMDdel52/*mdx* (4 copies) samples were used in all assays. Data normalization was done relative to copy number of *Mstn*, which was set to 2 copies in hDMDdel52/*mdx* mice. Compared to the *Mstn* copy number, inversion specific primers in assay E showed a double signal while assay F gave lower signals in hDMDdel52/*mdx* mice, while they did not give any signals in hDMD/*mdx* mice as expected.

Finally, we performed a more detailed analysis of the deleted exon 52 and its flanking introns. ddPCR analysis demonstrated that while assay B at the 5’ end of exon 52 was negative for all hDMDdel52/*mdx* and hDMDdel52-53/*mdx* mice, assay C at the 3’ end of exon 52 was unexpectedly positive with copy numbers identical to assay D (exon 53) (Fig 4). This suggested that hDMDdel52/*mdx* mice carry a partial deletion of exon 52. We thus sequenced the region around exon 52 of hDMDdel52/*mdx* mice (hDMDdel52/*mdx* mice were included in sequencing analysis and functional analyses) using Oxford Nanopore long read massive parallel sequencing (the sequencing data were deposited in the NCBI database with an accession number PRJNA630378). This revealed that there is a 2.3 kb inverted sequence originating from intron 51, which is located before the 5’ end of exon 52 and followed by exon 52 sequence starting from the 26^th^ nucleotide, indicating a deletion of the first 25 bps of exon 52 while the other 93 bps are still present. Additional primer pairs in this region were used and further confirmed this partial deletion/inversion event by Sanger sequencing. Since the splice acceptor site is deleted, this partial deletion at the DNA level leads to an exon 52 exclusion at the RNA level as reported previously [27]. Sequencing data also revealed that the partial exon 52 deletion occurred in both *hDMD* copies, while the intron 51 inversion did not show the same pattern. The most likely explanation would be that the inversion event did not occur in all copies of the *hDMD* gene. To further analyze the abundance of the intron 51 inversion we performed ddPCR with inversion specific primers (S2 Table). Primers were designed to specifically amplify the exon52/inversion (assay E) and the inversion/loxP/SV40 poly(A) (assay F) junction (Fig 7A).

All data was normalized to the copy number of *Mstn*, which should have 2 copies in hDMDdel52/*mdx* mice. As expected, the *hDMD* exon 1 copy number was 4 in homozygous hDMDdel52/*mdx* samples. Since each *hDMD* allele contains two copies of the *hDMD* gene, if the inversion was present in both copies, the expected copy number for the inversion specific probes would be 4 for homozygous hDMDdel52/*mdx* mice. While assay E (detecting the inversion/deletion junction) resulted in 4 copies, slightly lower copy numbers were observed in assay F (detecting the inversion/sv40 poly(A) junction). We attempted to elucidate the constitution of this area by Oxford Nanopore long read massive parallel sequencing. However, due to the inversion and duplication events, *de novo* assembly of this region based on short and long reads was unfeasible.

**Fig 8:**
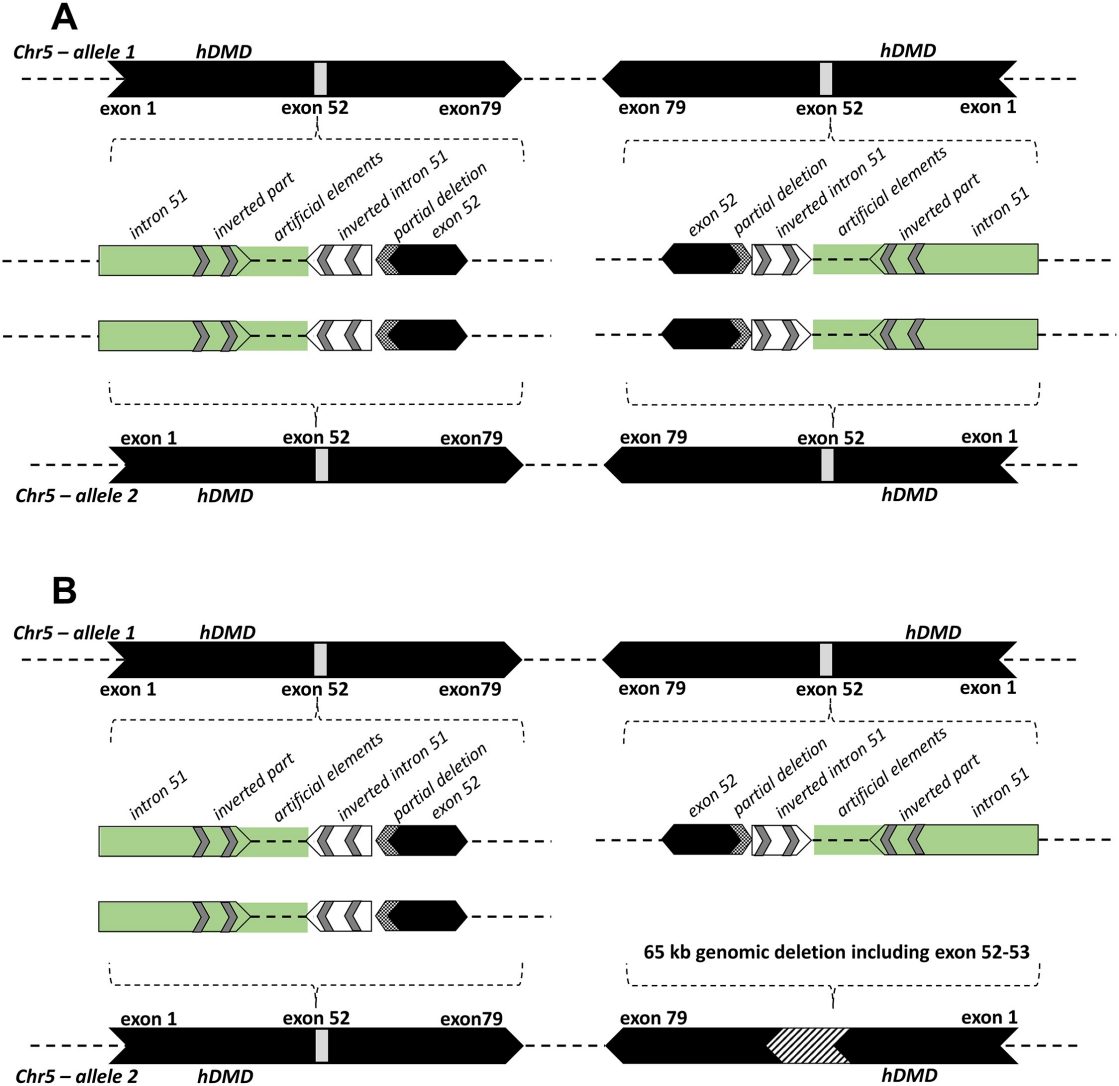
A schematic representation of *hDMD* gene constructs on mouse chromosome 5 of the hDMDdel52/*mdx* and hDMDdel52-53/*mdx* mice. A. The figure represents the tail-to-tail duplication of the *hDMD* gene, and the genetic rearrangements which include a partial deletion of exon 52 and an inversion of part of intron 51 in the hDMDdel52/*mdx* mouse. The green area including intron 51 and artificial elements could not be confirmed by sequencing. B. Because the *hDMD* gene in the hDMDdel52-53/*mdx* mouse was not sequenced, the schematic representation is mostly hypothetical but ddPCR results revealed that a ~65 kb genomic deletion including exon 52–53 (Fig 3) occurred in one *hDMD* copy and the other three copies remained intact.

In summary, detailed genetic analysis of hDMDdel52/*mdx* mice started with the observation of spontaneous dystrophin restoration in a cohort of mice (group 2), resulting from a ~65 kb genomic deletion including exon 52 and exon 53 which could be traced back to a single founder mouse. To elucidate the tandem duplication and genetic rearrangements around the deleted region, further experiments were carried out using hDMD/*mdx* and hDMDdel52/*mdx* (group 1) mice. Given the unexpected copy numbers observed during the analysis of this genomic region in hDMD/*mdx* and hDMDdel52/*mdx* mice, we combined FISH, sequencing and ddPCR analyses and showed that each *hDMDdel52* allele contains 2 copies of the *hDMD* gene, in a tail-to-tail orientation, where a partial deletion of exon 52 and an inversion of intron 51 are present in both *hDMD* gene copies (Fig 8A). Due to the complexity of the duplications and inversions, we are currently unable to construct a map spanning the whole region from intron 51 to intron 52 for each of the *hDMDdel52* copies. Furthermore, the ~65 kb genomic deletion in the hDMDdel52-53/*mdx* mice occurred only in one copy leaving three intact copies (Fig 8B).

### Overall gait is significantly affected in hDMDdel52/*mdx* mice

**Fig 9:**
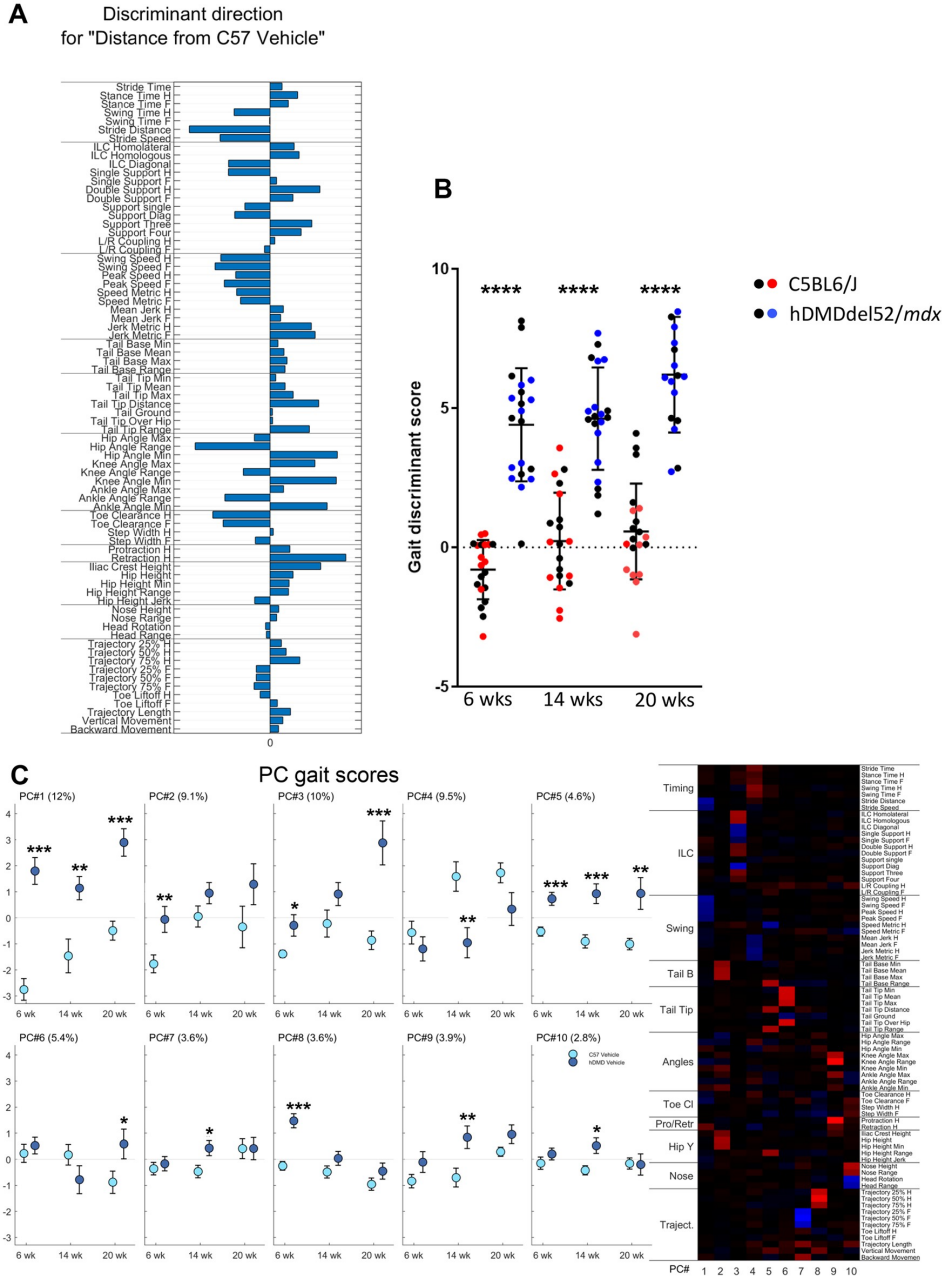
MotoRater analysis of hDMDdel52/*mdx* mice compared to C57BL6/J controls at the age of 6, 14 and 20 weeks. A. Discriminant vector representing an overall kinematic fingerprint of the differences between hDMDdel52/*mdx* and C57BL6/J mice. B. Gait discriminant score reflecting the totality of gait parameters. Black dots represent male mice in each group, while blue and red dots represent females. Statistical significance was assessed per time point using an unpaired two-tailed t-test (****: *P*<0.0001). Statistical differences were more pronounced in female mice. C. Heatmap showing 10 PC clusters reflecting different gait features that differ between hDMDdel52/*mdx* and C57BL6/J mice. The right panel displays the gait scores for the different PCs. The % of the total variation of the data explained by each PC is indicated between brackets. Values are presented as mean ± standard error of mean (SEM). Significance per time point was assessed using an unpaired two-tailed t-test (*: *P*<0.05; **: *P*<0.01; ***: *P*<0.001). hDMDdel52-53/*mdx* mice were not included in MotoRater analysis.

Despite the complex nature of the genotype of the hDMDdel52/*mdx* mice, the result is a dystrophin deficiency that is anticipated to result in a dystrophic phenotype. We recently described the highly sensitive MotoRater system to characterize motor function in *mdx* mice [33]. We here applied a similar analysis to quantify 95 gait scores in hDMDdel52/*mdx* and C57BL6/J controls at three different time points (age 6, 14 and 20 weeks). A discriminant vector was established based on those gait features, which demonstrated a large effect size. The vector can be seen as the overall kinematic fingerprint of the mice, characterizing all relevant gait differences of hDMDdel52/*mdx* mice compared to C57BL6/J controls (Fig 9A). This showed that the gait of hDMDdel52/*mdx* is highly different from healthy mice. Most striking differences included a shorter stride distance and slower overall speed, a changed inter-limb coordination (ILC) with less diagonal gait (trot) and more double support. In addition, hDMDdel52/*mdx* mice displayed decreased swing speeds but increased jerkiness and an increased overall hip height as well as increases in minimum hip, knee, and ankle joint angles.

An overall gait discriminant score was calculated, reflecting the totality of all gait changes and showed a highly significant difference (*P*<0.0001) between hDMDdel52/*mdx* mice and C57BL6/J controls at all time points (Fig 9B). Principal component (PC) analysis of the data allowed 10 PCs to be defined, representing different gait aspects (see heatmap, Fig 9C). PC1 reflected swing and stride speed and PC5 tail and hip height. Both PCs were significantly altered in hDMDdel52/*mdx* mice at all timepoints and together explained 16.6% of the total variation of data. Other PCs displayed significant differences at only one of the three time points: the 6 week time point in young mice (PC2 and PC8), the 14 week time point (PC4, PC7, PC9 and PC10) or at the 20 week time point (PC6) (Fig 8C).

## Discussion

AON-mediated splicing modulation is one of the therapeutic approaches currently in development for DMD, with three approved exon skipping drugs (eteplirsen, viltolarsen and golodirsen) and several others evaluated in clinical trials. However, next generation AONs applying different AON backbone modifications and/or conjugations are being explored to improve efficiency and/or delivery to muscle [34]. To allow a thorough screening of human specific AONs targeting exon 51 and exon 53 in a preclinical phase, we generated the hDMDdel52/*mdx* mouse model [27]. Thus far, only basic evaluations were performed of these mice, confirming the absence of dystrophin, a dystrophic phenotype in skeletal muscle and functional deficits in young animals using hanging wire tests. In this report we described a more detailed genetic and functional analysis of this model.

Using state-of-the-art and highly sensitive fine motor and gait kinematic analysis a strong and significant difference was evident in hDMDdel52/*mdx* mice at 6, 14 and 20 weeks of age, when compared to age- and gender-matched C57BL6/J controls. The main gait characteristics affected by the dystrophin absence in hDMDdel52/*mdx* mice included a shorter stride distance and slower overall speed, a changed inter-limb coordination with less diagonal gait (trot) and more double support. In addition, hDMDdel52/*mdx* mice displayed decreased swing speeds but increased jerkiness and an increased overall hip height as well as increases in minimum hip, knee, and ankle joint angles. Taken together, these changed gait parameters define a clear motor deficit in the hDMDdel52/*mdx* model that starts as early as 6 weeks and persists up to at least the age of 20 weeks. Some gait parameters displayed an age-dependent change that deteriorated with ageing, such as for example inter-limb coordination (PC3), while other differences with WT controls became less or even disappeared (PC7, PC8 and PC10), partially due to a shift in the gait of WT controls with increasing age. These data are in line with our previous data [33] showing a functional impairment in the hDMDdel52/*mdx* model using the forelimb grip strength test and two and four limb hanging tests [27], but show a much more pronounced motor and gait phenotype with a large enough window to allow accurate monitoring of therapeutic effects of AONs inducing exon 51 or 53 skipping in these mice in preclinical studies.

The genetics of the hDMDdel52/*mdx* mouse model was more complicated than initially anticipated. First, we discovered that both hDMD/*mdx* and hDMDdel52/*mdx* contain the *hDMD* gene in duplicate in a tail-to-tail orientation meaning the tail-to-tail duplication occurred during the generation of the original hDMD mouse This model was generated over 20 years ago, and has as far as we are aware not undergone any rearrangement of the transgene. In addition, the transgene in the hDMDdel52/*mdx* model appears relatively stable, since we have only observed the exon 53 deletion in a single founder mouse so far. The tail-to-tail duplication was not discovered during an earlier characterization of the hDMD/*mdx* model [26]. In that study COBRA FISH was used, to show a single integration site of the transgene in mouse chromosome 5. However, this technology did not have the resolution to detect the duplication of the transgene. It should be noted however, that the authors previously noticed that pyrosequence analysis, which was used for genotyping purposes, showed higher ratios of hDMD to *mdx* sequences than expected [26]. In hindsight these ratios fit with the duplication of the *hDMD* transgene. This duplication also explains why creating a deletion in heterozygous hDMD/*mdx* embryonic stem cells was extremely challenging using homologous recombination [27]. Thousands of targeted ES cells were selected based on carrying the antibiotic resistance gene that should replace the deleted exon, but then turned out not to carry the deletion. Upon reflection it is likely that the deletion in these cells was present, but on only one of the two copies.

The herein described hDMDdel52/*mdx* model was generated with the TALEN technology. What is interesting is that this introduced the exact same deletion of the first 25 bp of exon 52 in each of the two gene copies. The most likely scenario is that the nuclease generated a double-stranded break adjacent to exon 52 in each copy. Then the break was repaired with homologous recombination using the targeting vectors provided for one of the copies. Finally, the second break was repaired with homologous recombination using the repaired copy as a template. The partial deletion of exon 52 does not have an impact on exon skipping studies since exon 52 is not present on the mRNA level so that exon 51 or exon 53 skipping in each transcript produced from either gene copy can restore the reading frame. In addition, for genome editing approaches, dystrophin expression can be restored by editing of one copy or both. The only challenge is in generating additional mutations to be able to study other exons. It has been done successfully in the exon 52 [27] and exon 45 [35] deleted mouse models but having the additional copy makes generating deletions more difficult, since both gene copies have to be targeted. Notably, we also discovered additional genetic changes in this model, *i*.*e*. an inversion of intron 51 in each of the two gene copies. While this has no consequences for the model as such, it does underline that genome editing technologies like TALEN and CRISPR/Cas9 may cause also unintended changes at the target location. It is therefore important to characterize models created with genome editing techniques in great detail to confirm the intended genomic change and to also identify any additional editing events that may have occurred. Initially, we tried to amplify the deleted region by long range PCR, but we could not obtain the desired PCR products because of the inverted sequence and the complexity of this region. *hDMD* probes were then used to enrich the *hDMD* gene prior to Oxford Nanopore long read massive parallel sequencing. Using this technology, we were able to characterize the deletion/inversion event, however the artificial elements and inverted part of intron 51 that should have been in proximity of the deletion/inversion event were not picked up using the capture probes for this region, even though the theoretical distance should have been within the read length. This could be due to the design of the probes, which are not targeting artificial elements like SV40 poly(A) sequence, or because the event is more complex than we anticipated.

The third unexpected observation was the spontaneous restoration of dystrophin in a subset of the mice through a large deletion that removed both exons 52 and exon 53 and thus restored the reading frame in one of the two *hDMD* copies. This deletion event could be traced back to a single founder mouse and is likely due to a chance occurrence. In other colonies, containing hundreds of mice bred for over 10 generations, we have not observed anything similar yet. However, it is possible that due to the *hDMD* tail-to-tail duplication, secondary mutations can occur with a higher change during germ cell production. We thus recommend regular assessment of dystrophin expression in the hDMDdel52/*mdx* model to confirm its dystrophic phenotype. Breeding of the hDMDdel52-53/*mdx* mouse subpopulation has been terminated.

In conclusion, we provide a detailed genotypical and phenotypical characterization of the hDMDdel52/*mdx* model that confirms its usefulness for the preclinical screening of DMD drug candidates.

## Supporting information

S1 Fig Sanger sequencing analysis of exon 52 deleted region. Sanger sequencing results confirms the junctions of exon 52/intron 51 inversion (A) and intron 51 inversion/sv40 poly (A) signal (B). Red lines represent the sequenced regions.
(TIF)

S1 Table Probes and primers used for TaqMan analysis. (DOCX)

S2 Table Primers used for EvaGreen-based ddPCR. (DOCX)

## References

[1] Moat SJ, Bradley DM, Salmon R, Clarke A, Hartley L. Newborn bloodspot screening for Duchenne muscular dystrophy: 21 years experience in Wales (UK). European journal of human genetics: EJHG. 2013;21(10):1049–53. Epub 2013/01/24. 10.1038/ejhg.2012.301 23340516 PMC3778339

[2] Gao QQ, McNally EM. The Dystrophin Complex: Structure, Function, and Implications for Therapy. Compr Physiol. 2015;5(3):1223–39. Epub 2015/07/04. 10.1002/cphy.c140048 26140716 PMC4767260

[3] Rader EP, Turk R, Willer T, Beltrán D, Inamori K-i, Peterson TA, et al Role of dystroglycan in limiting contraction-induced injury to the sarcomeric cytoskeleton of mature skeletal muscle. 2016;113(39):10992–7. 10.1073/pnas.1605265113 %J Proceedings of the National Academy of Sciences. 27625424 PMC5047148

[4] Pane M, Scalise R, Berardinelli A, D’Angelo G, Ricotti V, Alfieri P, et al Early neurodevelopmental assessment in Duchenne muscular dystrophy. Neuromuscular disorders: NMD. 2013;23(6):451–5. Epub 2013/03/29. 10.1016/j.nmd.2013.02.012 . 23535446

[5] Shimizu-Motohashi Y, Miyatake S, Komaki H, Takeda S, Aoki Y. Recent advances in innovative therapeutic approaches for Duchenne muscular dystrophy: from discovery to clinical trials. American journal of translational research. 2016;8(6):2471–89. Epub 2016/07/12. 27398133 PMC4931144

[6] Muntoni F, Wood MJ. Targeting RNA to treat neuromuscular disease. Nature reviews Drug discovery. 2011;10(8):621–37. Epub 2011/08/02. 10.1038/nrd3459 . 21804598

[7] Muntoni F, Torelli S, Ferlini A. Dystrophin and mutations: one gene, several proteins, multiple phenotypes. The Lancet Neurology. 2003;2(12):731–40. Epub 2003/11/26. 10.1016/s1474-4422(03)00585-4 . 14636778

[8] Hendriksen RG, Hoogland G, Schipper S, Hendriksen JG, Vles JS, Aalbers MW. A possible role of dystrophin in neuronal excitability: a review of the current literature. Neurosci Biobehav Rev. 2015;51:255–62. Epub 2015/02/14. 10.1016/j.neubiorev.2015.01.023 . 25677308

[9] Aartsma-Rus A, Van Deutekom JC, Fokkema IF, Van Ommen GJ, Den Dunnen JT. Entries in the Leiden Duchenne muscular dystrophy mutation database: an overview of mutation types and paradoxical cases that confirm the reading-frame rule. Muscle & nerve. 2006;34(2):135–44. Epub 2006/06/14. 10.1002/mus.20586 . 16770791

[10] Bladen CL, Salgado D, Monges S, Foncuberta ME, Kekou K, Kosma K, et al The TREAT-NMD DMD Global Database: analysis of more than 7,000 Duchenne muscular dystrophy mutations. Human mutation. 2015;36(4):395–402. Epub 2015/01/22. 10.1002/humu.22758 25604253 PMC4405042

[11] Maruyama R, Yokota T. Creation of DMD Muscle Cell Model Using CRISPR-Cas9 Genome Editing to Test the Efficacy of Antisense-Mediated Exon Skipping. Methods Mol Biol. 2018;1828:165–71. Epub 2018/09/02. 10.1007/978-1-4939-8651-4_10 . 30171541

[12] Akpulat U, Wang H, Becker K, Contreras A, Partridge TA, Novak JS, et al Shorter Phosphorodiamidate Morpholino Splice-Switching Oligonucleotides May Increase Exon-Skipping Efficacy in DMD. Mol Ther Nucleic Acids. 2018;13:534–42. Epub 2018/11/06. 10.1016/j.omtn.2018.10.002 30396145 PMC6222172

[13] Andrews JG, Wahl RA. Duchenne and Becker muscular dystrophy in adolescents: current perspectives. Adolesc Health Med Ther. 2018;9:53–63. Epub 2018/03/29. 10.2147/AHMT.S125739 29588625 PMC5858539

[14] Nakamura A, Fueki N, Shiba N, Motoki H, Miyazaki D, Nishizawa H, et al Deletion of exons 3–9 encompassing a mutational hot spot in the DMD gene presents an asymptomatic phenotype, indicating a target region for multiexon skipping therapy. Journal of human genetics. 2016;61(7):663–7. Epub 2016/03/25. 10.1038/jhg.2016.28 . 27009627

[15] Bello L, Morgenroth LP, Gordish-Dressman H, Hoffman EP, McDonald CM, Cirak S. DMD genotypes and loss of ambulation in the CINRG Duchenne Natural History Study. Neurology. 2016;87(4):401–9. Epub 2016/06/28. 10.1212/WNL.0000000000002891 27343068 PMC4977110

[16] Bello L, Pegoraro E. Genetic diagnosis as a tool for personalized treatment of Duchenne muscular dystrophy. Acta myologica: myopathies and cardiomyopathies: official journal of the Mediterranean Society of Myology. 2016;35(3):122–7. Epub 2017/05/10. 28484312 PMC5416739

[17] Aartsma-Rus A, Fokkema I, Verschuuren J, Ginjaar I, van Deutekom J, van Ommen G-J, et al Theoretic applicability of antisense-mediated exon skipping for Duchenne muscular dystrophy mutations. 2009;30(3):293–9. 10.1002/humu.20918 19156838

[18] Lim KR, Maruyama R, Yokota T. Eteplirsen in the treatment of Duchenne muscular dystrophy. Drug design, development and therapy. 2017;11:533–45. Epub 2017/03/11. 10.2147/DDDT.S97635 28280301 PMC5338848

[19] Aartsma-Rus A, Arechavala-Gomeza V. Why dystrophin quantification is key in the eteplirsen saga. Nature reviews Neurology. 2018;14(8):454–6. Epub 2018/07/04. 10.1038/s41582-018-0033-8 . 29967362

[20] Aartsma-Rus A, Corey DR. The 10th Oligonucleotide Therapy Approved: Golodirsen for Duchenne Muscular Dystrophy. Nucleic Acid Ther. 2020 Epub 2020/02/12. 10.1089/nat.2020.0845 . 32043902 PMC7133412

[21] Aartsma-Rus A, Goemans N. A Sequel to the Eteplirsen Saga: Eteplirsen Is Approved in the United States but Was Not Approved in Europe. Nucleic Acid Ther. 2019;29(1):13–5. Epub 2018/12/12. 10.1089/nat.2018.0756 . 30526286

[22] Dhillon S. Viltolarsen: First Approval. Drugs. 2020;80(10):1027–31. Epub 2020/06/11. 10.1007/s40265-020-01339-3 . 32519222

[23] FDA NEWS RELEASE. FDA Approves Targeted Treatment for Rare Duchenne Muscular Dystrophy Mutation. 2020. https://www.fda.gov/news-events/press-announcements/fda-approves-targeted-treatment-rare-duchenne-muscular-dystrophy-mutation.

[24] McGreevy JW, Hakim CH, McIntosh MA, Duan D. Animal models of Duchenne muscular dystrophy: from basic mechanisms to gene therapy. 2015;8(3):195–213. 10.1242/dmm.018424 %J Disease Models & Mechanisms. 25740330 PMC4348559

[25] Sicinski P, Geng Y, Ryder-Cook A, Barnard E, Darlison M, Barnard P. The molecular basis of muscular dystrophy in the mdx mouse: a point mutation. 1989;244(4912):1578–80. 10.1126/science.2662404 %J Science. 2662404

[26] t Hoen PA, de Meijer EJ, Boer JM, Vossen RH, Turk R, Maatman RG, et al Generation and characterization of transgenic mice with the full-length human DMD gene. J Biol Chem. 2008;283(9):5899–907. Epub 2007/12/18. 10.1074/jbc.M709410200 . 18083704

[27] Veltrop M, van Vliet L, Hulsker M, Claassens J, Brouwers C, Breukel C, et al A dystrophic Duchenne mouse model for testing human antisense oligonucleotides. PLoS One. 2018;13(2):e0193289 Epub 2018/02/22. 10.1371/journal.pone.0193289 29466448 PMC5821388

[28] Veltrop M, van der Kaa J, Claassens J, van Vliet L, Verbeek S, Aartsma-Rus A. Generation of embryonic stem cells and mice for duchenne research. PLoS Curr. 2013;5 Epub 2013/09/24. 10.1371/currents.md.cbf1d33001de80923ce674302cad7925 24057032 PMC3775890

[29] Beekman C, Janson AA, Baghat A, van Deutekom JC, Datson NA. Use of capillary Western immunoassay (Wes) for quantification of dystrophin levels in skeletal muscle of healthy controls and individuals with Becker and Duchenne muscular dystrophy. PLoS One. 2018;13(4):e0195850 Epub 2018/04/12. 10.1371/journal.pone.0195850 29641567 PMC5895072

[30] Livak KJ, Schmittgen TD. Analysis of relative gene expression data using real-time quantitative PCR and the 2(-Delta Delta C(T)) Method. Methods. 2001;25(4):402–8. Epub 2002/02/16. 10.1006/meth.2001.1262 . 11846609

[31] Verheul RC, van Deutekom JC, Datson NA. Digital Droplet PCR for the Absolute Quantification of Exon Skipping Induced by Antisense Oligonucleotides in (Pre-)Clinical Development for Duchenne Muscular Dystrophy. PLoS One. 2016;11(9):e0162467 Epub 2016/09/10. 10.1371/journal.pone.0162467 27612288 PMC5017733

[32] Gazzoli I, Pulyakhina I, Verwey NE, Ariyurek Y, Laros JF, t Hoen PA, et al Non-sequential and multi-step splicing of the dystrophin transcript. RNA biology. 2016;13(3):290–305. Epub 2015/12/17. 10.1080/15476286.2015.1125074 26670121 PMC4829307

[33] Datson NA, Bijl S, Janson A, Testerink J, van den Eijnde R, Weij R, et al Using a State-of-the-Art Toolbox to Evaluate Molecular and Functional Readouts of Antisense Oligonucleotide-Induced Exon Skipping in mdx Mice. Nucleic Acid Ther. 2020;30(1):50–65. Epub 2019/12/11. 10.1089/nat.2019.0824 31821107 PMC7049912

[34] Cell-Penetrating Peptide Conjugates of Steric Blocking Oligonucleotides as Therapeutics for Neuromuscular Diseases from a Historical Perspective to Current Prospects of Treatment. 2019;29(1):1–12. 10.1089/nat.2018.0747 . 30307373 PMC6386087

[35] Young CS, Mokhonova E, Quinonez M, Pyle AD, Spencer MJ. Creation of a Novel Humanized Dystrophic Mouse Model of Duchenne Muscular Dystrophy and Application of a CRISPR/Cas9 Gene Editing Therapy. J Neuromuscul Dis. 2017;4(2):139–45. Epub 2017/05/17. 10.3233/JND-170218 28505980 PMC5565771

