# Supplementary figures and images for: Detailed genetic and functional analysis of the hDMDdel52/mdx mouse model
Source: PLoS One. 2020 Dec 23;15(12):e0244215. doi: 10.1371/journal.pone.0244215 (PMC7757897; doi:10.1371/journal.pone.0244215)

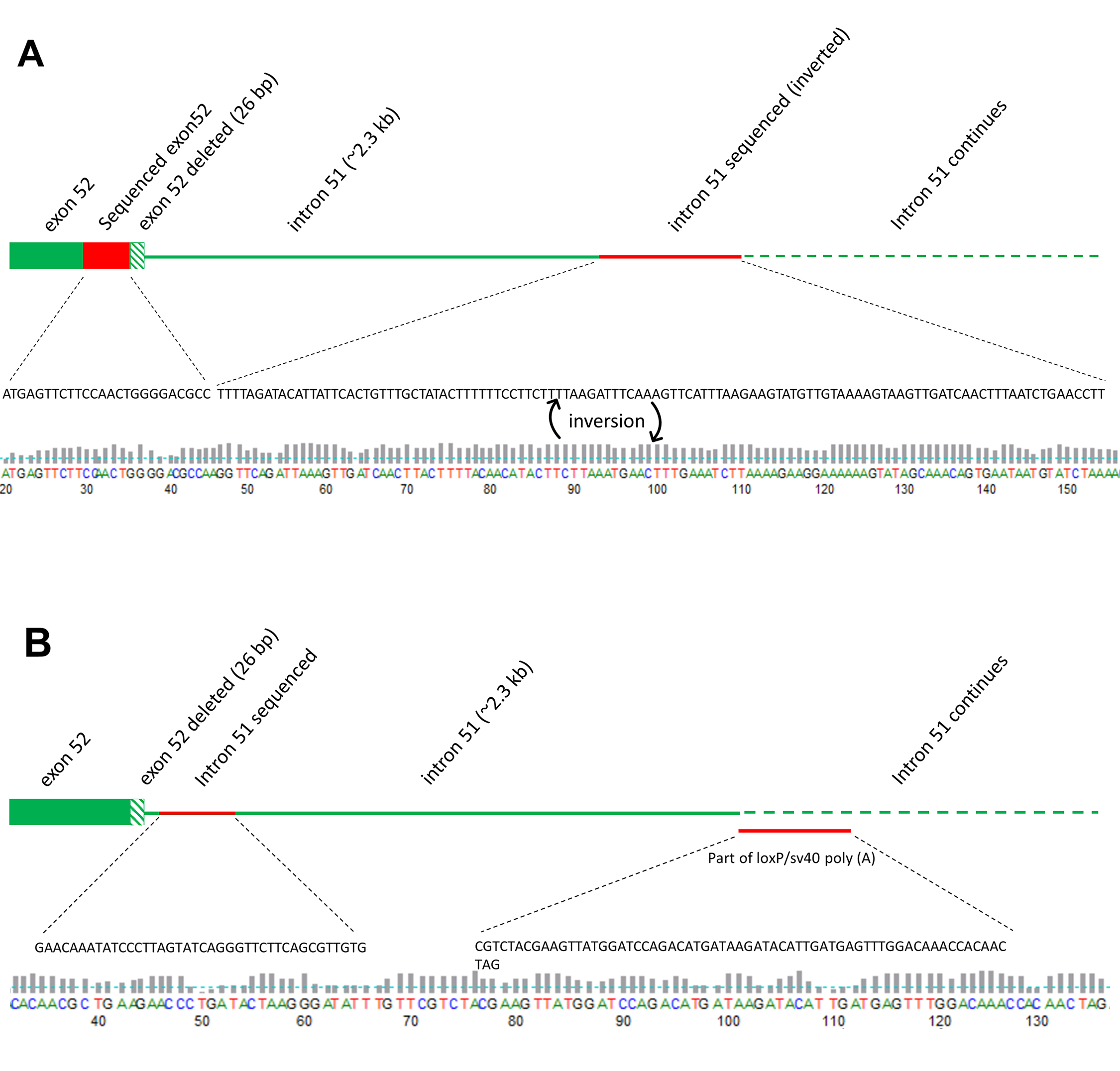

Supplement: S1 Fig — Sanger sequencing results confirms the junctions of exon 52/intron 51 inversion (A) and intron 51 inversion/sv40 poly (A) signal (B). Red lines represent the sequenced regions. (TIF) [file pone.0244215.s001.tif]
