# Supplementary material for: Detailed genetic and functional analysis of the hDMDdel52/mdx mouse model
Source: PLoS One. 2020 Dec 23;15(12):e0244215. doi: 10.1371/journal.pone.0244215 (PMC7757897; doi:10.1371/journal.pone.0244215)
Supplement: S1 Table — (DOCX) [file pone.0244215.s002.docx]

**S1 Table.** **Probes and primers used for TaqMan analysis**

| **Assay Name** | **Target** | **Forward Primer** | **Reverse Primer** | **Probe** |
| --- | --- | --- | --- | --- |
| DMD_e51-i51 (Assay A) | exon 51 - intron 51 | CCTTGAGGATATCAACGAGATGATC | GAGTAAAGTGATTGGTGGAAAATCTTC | TCAAGCAGAAGGTATGAGAA |
| DMD_i51-e52 (Assay B) | intron 51 - exon 52 | AACGCTGAAGAACCCTGATACTAAG | GACGCCTCTGTTCCAAATCC | TCTTACAGGCAACAATG |
| DMD_e52-i52 (Assay C) | exon 52 - intron 52 | GCAATCAAGAGGCTAGAACAATCA | CTTTGTGTGTCCCATGCTTGTTA | TACGGATCGAAGTAAGTTT |
| DMD_i52-e53 (Assay D) | intron 52 - exon 53 | TGTCTCCTCCAGACTAGCATTTACTACT | GTGTTCTTGTACTTCATCCCACTGA | TTCTAGTTGAAAGAATTCAG |
| hDMD_e73-i73 | exon 73 - intron 73 | AATGGAAAACAGCAATGGATCTTAT | AAAGCAATTTCATTGTCAGGAACA | CTCCTAATGAGAGCATGTAAGTA |
| hDMD_i73-e74 | intron 73 - exon 74 | GTCCCTAACCCCCAAAGCAA | GGTTCAAACTTTGGCAGTAATGC | CCAGAGATGATGAACATT |
| hDMD_e75-i75 | exon 75 - intron 75 | AAACAGCTGGAGTCACAGTTACACA | GTAAAAATCCCATCTCTCTCCTCACT | AAGGCAGCTGCTGGA |
| hDMD_e76-i76 | exon 76 - intron 76 | CGAGTGGTTGGCAGTCAAACT | AGACAACAAAATCTGAGAGTAGCTAGGA | CGGACTCCATGGGTAA |
| hDMD_e77-i77 | exon 77 - intron 77 | CTCAACAACTCCTTCCCTAGTTCAA | CCAGCAAATCTGAGTCCCTTCT | AGGTAAGCTCCAATACC |
| hDMD_e78-i78 | exon 78 - intron 78 | AAAGGAAGAAATACCCCTGGAAA | TGGCCGTGAGCCTGAATC | CCAATGAGAGAGGTTAGTG |
| hDMD_i78-e79 | intron 78 - exon 79 | TGATGCTATCTATCTGCACCTTTTG | GACTTCCTACATTGTGTCCTGGAA | AAAGTCTGTCTTTCTTTCTCT |
| hDMD_ex79 3'utr | exon 79- exon 79 utr | GGAAACTACACCACACTAAAACATTGTC | TCGTTGTCAGTGGAAAGTTGTTTAA | ACAGCTCCAGATGTTT |
