# Supplementary material for: Detailed genetic and functional analysis of the hDMDdel52/mdx mouse model
Source: PLoS One. 2020 Dec 23;15(12):e0244215. doi: 10.1371/journal.pone.0244215 (PMC7757897; doi:10.1371/journal.pone.0244215)
Supplement: S2 Table — (DOCX) [file pone.0244215.s003.docx]

**S2 Table**. **Primers used for EvaGreen-based ddPCR**

| **Assay Name** | **Target** | **Forward Primer** | **Reverse Primer** |
| --- | --- | --- | --- |
| DMD_inv51_e52 (Assay E) | e52/inv51 | CAGTTTTAGATACATTATTCACTGTTTGC | CTAGCCTCTTGATTGCTGGTCT |
| DMD_sv40_inv51 (Assay F) | Inv51/sv40 | GCATTCTAGTTGTGGTTTGTCCA | AGTGTTTTGGCTGGTCTCACA |
| hDMD exon 1 | *hDMD* exon 1 | TCTACTTCTTCCCACCAAAGCAT | AAGCTGCTGAAGTTTGTTGGTT |
| *Mstn* | *Mstn* | CTCAGACCCGTCAAGACTCC | CCTGGGCTCATGTCAAGTTT |
